# Effects of Flavonoids Extracted from *Citrus aurantium* on Performance, Behavior, and Rumen Gene Expression in Holstein Bulls Fed with High-Concentrate Diets in Pellet Form

**DOI:** 10.3390/ani11051387

**Published:** 2021-05-13

**Authors:** Montserrat Paniagua, Francisco Javier Crespo, Anna Arís, Maria Devant

**Affiliations:** 1 Ruminant Production, IRTA (Institut de Recerca i Tecnologia Agroalimentàries), Torre Marimon, Caldes de Montbui, 08140 Barcelona, Spain; mpaniagua@quimidroga.com (M.P.); anna.aris@irta.cat (A.A.); 2 Quimidroga SA, 08006 Barcelona, Spain; 3 HealthTech Bio Actives, S.L.U., 08029 Barcelona, Spain; jcrespo@htba.com

**Keywords:** bulls, flavonoids, behavior, rumen inflammation, bitter taste receptors

## Abstract

**Simple Summary:**

Currently, the beef production system faces important challenges, such as improving feed efficiency, reducing environmental impact, and improving animal welfare. Citrus flavonoids from bitter orange plant secondary metabolites are feed additives that have shown promising effects on intake modulation, efficiency, and improving animal behaviors related with animal welfare. However, as they interact with the digestive tract microbiota and the digestive tract receptors, their effects may be affected by the feeding method (mash or pellet). In the present study, when these flavonoids were fed in a pellet concentrate presentation form, the bull’s efficiency did not improve. However, animal behaviors related to welfare problems were reduced. Furthermore, supplementing bulls with flavonoids modified the expression in the rumen of genes concerned with behavior and inflammatory response. Therefore, supplementing bulls with citrus flavonoids may be a good strategy to improve their welfare.

**Abstract:**

Flavonoid supplementation may modify the behavior and rumen inflammatory response of fattening bulls, and this could be related to the concentrate presentation (mash or pellet) form. In the present study, 150 Holstein bulls (183.0 ± 7.53 kg BW and 137 ± 1.8 d of age) were randomly allocated to one of eight pens and assigned to control (C) or (BF) (*Citrus aurantium*, Bioflavex CA, HealthTech Bio Actives, Spain, 0.4 kg per ton of concentrate of Bioflavex CA, 20% naringin). Concentrate (pellet) intake was recorded daily, and BW and animal behavior fortnightly. Animals were slaughtered after 168 d of study, and ruminal epithelium samples were collected for gene expression analyses. Treatment did not affect animal performance; however, BF supplementation reduced agonistic interactions and oral non-nutritive behaviors and increased the time devoted to eating concentrate and ruminating activity (*p* < 0.05). The gene expression of some genes in the rumen epithelium was greater or tended to be greater in BF than C bulls (*bitter taste receptor 16*, cytokine *IL-25*, *β-defensin*; *p* < 0.10; *pancreatic polypeptide receptor 1* and *tumor necrosis factor alpha*; *p* < 0.05). In conclusion, flavonoid supplementation modifies the expression of genes in the rumen epithelium that could be related to inflammation and animal behavior modulation.

## 1. Introduction

In recent years, different plant secondary metabolites in beef cattle to improve animal health, productivity, and efficiency have shown promising results as natural alternatives to chemicals, drugs, and growth promoters [1]. The exact mode of action of these compounds remains unknown. Citrus flavonoids may affect rumen microbiota and fermentation [2,3] or might directly interact with several receptors in the rumen, modifying eating and animal behavior in bulls fed high-concentrate diets [4,5]. Additionally, Paniagua et al. [5] fed bulls a high-concentrate diet in meal presentation form and found that citrus flavonoids reduced the gene expression of the bitter taste receptors (TAS2R) analyzed in the rumen epithelium of supplemented bulls. Therefore, it was hypothesized that citrus flavonoids might modulate the eating pattern in bulls, acting over these TAS2Rs and modifying the release of hormones and peptides involved in hunger and satiety. Finally, Paniagua et al. [4,5] found that citrus flavonoid supplementation modified animal behavior in bulls, reducing the sexual and agonistic interactions studied and increasing the time devoted to eating as well. Thus, it was hypothesized that inflammation in the ruminal epithelium might be involved in animal behavior modulation through mechanisms related to the gut–brain axis [5]. The concentrate presentation (pellet vs. meal) modulates the eating pattern of the animals, rumen fermentation, and genes related to eating and animal behavior [6]; therefore, the presentation form might affect naringin ruminal metabolism and its impact on eating and animal behavior.

Accordingly, the present study was designed to explore the effects of citrus flavonoid supplementation in bulls fed high-concentrate diets in pellet form on performance (concentrate consumption, growth, and concentrate efficiency), carcass characteristics, rumen wall health, and animal behavior in commercial conditions. Additionally, the expression of genes involved in the gut–brain axis crosstalk, such as bitter taste receptors, some neurotransmitters receptors, and different inflammation regulators, were investigated in rumen epithelium to highlight the mechanisms involved in eating and animal behavior modulation when bulls are supplemented with citrus flavonoids.

## 2. Materials and Methods

### 2.1. Animals, Feeding, Housing, and Experimental Design

This study was conducted in accordance with the Spanish guidelines for experimental animal protection (Royal Decree 53/2013 of 1 February on the protection of animals used for experimentation or other scientific purposes; Boletín Oficial del Estado, 2013). One hundred fifty Holstein bulls (183.0 ± 7.53 kg of body weight (BW) and 137 ± 1.8 d of age) were fattened under commercial conditions on a farm (Granja l’Alsina, L’Alsina, Lleida, Spain). The whole study lasted 168 d and was divided into growing (0 to 112 d) and finishing (113 to 168 d) phases. Animals were randomly allocated to one of eight pens and assigned to one of the two treatments (4 pens per treatment and 18 animals per pen): either control (C) or supplemented (BF) with 0.04% of bitter orange extract (*Citrus aurantium*) of the whole fruit rich in naringin (>20%) (Bioflavex CA, HealthTech Bio Actives, Barcelona, Spain). Bioflavex was incorporated into the concentrate during the concentrate manufacturing. The pelleting process started with a grinding process of the ingredients through a roller mill with 2.75 mm sized screen openings as described by Verdú et al. [7]. The manufactured pellets had a uniform diameter (3.5 mm) and length (10 mm). 

The pens were totally covered (12 m × 6 m) and were deep bedded with straw and equipped with a three-space feeder (1.50 m length, 0.40 m width, 1.50 m height, and 0.35 m depth). The feeder of each pen weighed the concentrate continuously as described by Verdú et al. [7], and these data were recorded to calculate the concentrate consumption by pen. Pens were also equipped with one drinker (0.30 m length, 0.30 m width, 0.18 m depth). Straw was offered ad libitum in a separated straw five-space feeder (3.60 m length, 1.10 m wide, and 0.32 m depth), and every time it was replaced, it was recorded to estimate the total straw consumption. As straw was also used for bedding, these data are only an estimation. 

### 2.2. Feed Consumption and Performance

Animals were fed a commercial concentrate in meal form, formulated to cover their nutritional requirements [8]. Ingredients and nutritional composition of the concentrates were the same as in a previous study conducted on the same farm with a concentrate in a meal presentation form [5]. During the first 112 d of the study, animals were fed a growing concentrate, and between 112 d and the end of the study, animals were fed a finishing concentrate (Table 1). Throughout the study, animals had ad libitum access to wheat straw (3.5% CP, 1.6% ether extract, 70.9% NDF, and 6.1% ashes; DM basis) and fresh water.

Animals were weighed individually every 14 d throughout the study in 12 experimental periods of 14 d. As previously mentioned, during the first 8 periods (from 1 d to 112 d), the animals consumed the growing concentrate, and during the last 4 periods (from 113 d to 168 d), as well as during the days before slaughter, the animals consumed the finishing concentrate. After 168 d of study, the bulls were transported to the slaughterhouse (Escorxador del Grup Alimentari Guissona, Guissona, Spain), located 15 km from the farm. Animals were slaughtered in 2 weeks, four pens per week, two pens from the C and two from the BF bulls each week. The time spent waiting before slaughter was less than 6 h. Animals were weighed before loading. They were slaughtered by commercial practices and following the EU Regulation 1099/2009 on the protection of animals at the time of killing or slaughtering.

### 2.3. Animal Behavior

A visual scan procedure at days 13, 28, 44, 56, 72, 83, 100, 114, 128, 143, 153, and 167 of the study was performed to study the general activity (standing, lying, eating, drinking, and ruminating) and social behavior (nonagonistic, agonistic, and sexual interactions) of the animals in every pen. Nonagonistic interacions included self-grooming, social behavior, and oral non-nutritive behaviors. Agonistic behaviors included butting, displacement, chasing, and chasing up. Finally, sexual interactions included flehmen, attempted mounts, and completed mounts. The social behavior and general activities studied were recorded as described by Paniagua et al. [4,5]. The visual observation was made for 2 pens at the same time from 8:00 to 10:30 am. General activities were scored using 3 scan samplings of 10 s at 5 min intervals, and social behavior was scored during three continuous sampling periods of 5 min. This scanning procedure of 15 min was repeated twice consecutively in each pen, starting randomly in a different pen every scanning day.

### 2.4. Carcass Quality

After slaughtering, hot carcass weight (HCW) was registered for every animal. Dressing percentage was calculated by dividing the HCW by the BW recorded before slaughtering. Following the (S)EUROP categories described by EU Regulations No. 1208/81 and No. 1026/91, the conformation of carcasses was classified, where “E” corresponded to an excellent conformation, “U” to a very good conformation, “R” to a good conformation, “O” to a fair conformation, and “P” to a poor conformation. The fat cover was classified according to EU Regulation No. 1208/81, which utilizes a classification system by numbers (1, 2, 3, 4, 5), where 5 corresponds to a very high degree of covering fat and heavy fat deposits in the thoracic cavity and 1 is classified as a low degree, with no fat cover.

### 2.5. Rumen and Liver Macroscopic Evaluation and Sample Collection

The rumen and liver of every animal were macroscopically evaluated at the slaughterhouse. Rumens were classified based on color by a visual evaluation, from 1 to 5, “5” being a black-colored rumen, and “1” being a white-colored rumen [9]. They were also divided into areas according to Lesmeister et al. [10] to examine the presence of ulcers, baldness regions, and clumped papillae [11]. Liver abscesses were classified according to Brown et al. [12].

Additionally, a liquid sample from rumen was obtained from homogeneous contents strained through a cheesecloth from 18 animals randomly selected from two pens per treatment, immediately following slaughter. Following the procedures of Jounay [13], 4 mL of ruminal fluid was mixed with 1 mL of a solution containing 0.2% (*wt/wt*) mercuric chloride, 2% (*wt/wt*) orthophosphoric acid, and 2 mg/mL of 4-methylvaleric acid (internal standard) in distilled water and stored at −20 °C until subsequent volatile fatty acids (VFA) analysis. Also, a 1-cm^2^ section of the rumen wall (left side of the cranial ventral sac) was sampled, and papillae were excised before being rinsed 2 times with chilled PBS after sampling and then immediately incubated in RNAlater (Invitrogen, Madrid, Spain) to preserve the RNA integrity. After 24 h of incubation with RNAlater at 4 °C, the liquid was removed, and tissue was frozen at −80 °C until further RNA extraction and subsequent gene expression analysis.

### 2.6. Biological and Chemical Analyses

During the study, samples of concentrate were collected at 0, 42, 84, 126, and 168 d and analyzed for DM (by method 925.04 [14]), ash (by method 642.05 [14]), CP (by the Kjeldahl method, method 988.05 [14]), ADF and NDF (according to Van Soest et al. [15], using sodium sulfite and alpha-amylase), and ether extract (EE) (by Soxhlet with a previous acid hydrolysis, method 920.39 [14]).

Naringin was determined for every sample of concentrate (C and BF) as a Bioflavex CA marker for BF group and was used as a marker confirming the adequate inclusion of citrus flavonoid extract in the diets by Laboratory of HealthTech Bio Actives. An internal method for naringin quantification using HLPC developed by HealthTech Bio Actives was used [4].

Ruminal VFA concentration was determined with a semicapillary column (15 m × 0.53 mm ID, 0.5 µm film thickness, TRB-FFAP, Teknokroma, Barcelona, Spain) composed of 100% polyethylene glycol (PEG) esterified with nitroterephtalic acid, bonded and crosslinked phase (method number 5560; APHA–AWWA–WPCF, 2005), using a CP-3800-GC (Varian, Inc., Walnut Creek, CA, USA). Ruminal liquid pH was immediately measured at the slaughterhouse with a portable pH meter (Crison pH25, Crison Instruments SA, Barcelona, Spain). 

For gene expression analyses, the total RNA was extracted from rumen wall homogenizing tissues in Trizol (Invitrogen, Waltham, MA, USA) by Polytron Instrument (IKA, Staufen, Germany). Isolated mRNA was reverse transcribed to cDNA using a PrimeScript RT Reagent Kit (Takara, Frankfurt, Germany) following the manufacturer’s instructions. The RNA purity was assessed by a NanoDrop instrument (ThermoFisher, Madrid, Spain) at 260, 280, and 230 nm. The quantification of the expression of genes at the mRNA level coding for (1) the tight junction protein Claudin4 (CLDN4); (2) the production, expression, and turnover of neurotransmitters: free fatty acid receptor 2 (ffr2) and free fatty acid receptor 3 (ffr3), pancreatic polypeptide receptor 1 (ppyr1), actual name neuropeptide Y receptor Y4 [npy4r]), α2-adrenergic receptor subtype C (adra2c), and cholecystokinin receptor 4 (cckbr); (3) pro-inflammatory cytokines TNF-α (TNFα) and cytokine IL-25 (IL-25), pattern recognition receptor Toll-like receptor 4 (TLR4), and antimicrobial peptides released by intestinal cells (β-defensins and lactoferrin); (4) bitter taste receptors type 2 member 7, 16, 38, and 39 (TAS2R7, TAS2R16, TAS2R38 and TAS2R39) was performed by quantitative PCR (qPCR). The qPCR was performed as described in Paniagua et al. [5].

### 2.7. Calculations and Statistical Analyses

Only the pen was considered the experimental unit, and the animals within the pen were considered sampling units in some parameters.

The coefficient of variation (CV) of average daily gain (ADG) and BW was calculated as the standard deviation of the individual data of the animals in a pen in a 14-d period divided by the mean of these data. The coefficient of variation (CV) of intake was calculated as the standard deviation of the data of an animal in a 14-d period divided by the mean of these data. The CV of the concentrate efficiency data were transformed into a log to achieve a normal distribution. The means presented in the tables and figures correspond to nontransformed data, and SEM and *p*-values correspond to the ANOVA analyses of the transformed data. The percentage of each general activity was calculated, and the averages by day, pen, and scan obtained. Then, these data were transformed into natural logarithms to achieve a normal distribution. The frequency of each social behavior was calculated by summing by day, pen, and scan and transformed into the root of the sum of each activity plus 1 to achieve a normal distribution. The ANOVA analysis was performed with transformed data, and the means shown in the tables correspond to the back transformed data. 

Unification of performance, animal behavior, and concentrate consumption data averaged by pen and period were analyzed using a mixed effects model (Version 9.2, SAS Inst., Inc., Cary, NC, USA). The model included the initial BW as a covariate treatment period (14-d period), the interaction between treatment and period as fixed effects, and the pen as a random effect. The period was considered a repeated factor, and for each analyzed variable, the animal nested within the interaction between treatment and pen (the error term) was subjected to 3 variance–covariance structures: compound symmetry, autoregressive order one, and unstructured. The covariance structure that yielded the smallest Schwarz’s Bayesian information criterion was considered the most desirable analysis.

In the case of rumen gene expression, data were transformed into a log to achieve a normal distribution. The means presented in the figure correspond to nontransformed data, and SEM and *p*-values correspond to the ANOVA analyses of the transformed data. Pens were considered the experimental unit and animals the sampling units, and data were analyzed using ANOVA where the model included treatment (as there were no repeated measures) as the main effect. For VFA and pH data, pens were also considered the experimental unit and animals the sampling units, and data were analyzed using ANOVA where the model included treatment (as there were no repeated measures) as the main effect. HCW was analyzed using a mixed effects model (Version 9.2, SAS Inst., Inc., Cary, NC, USA) including initial BW as a covariate, the treatment as a fixed effect, and the pen as a random effect. For categorical variables analyses (carcass classification, rumen health parameters, hepatic abscesses) a chi-square test was used. Differences were declared significant at *p* < 0.05, and trends were discussed at 0.05 ≤ *p* ≤ 0.10 for all models. Significances were indicated as follows: *** = *p* < 0.001; ** = *p* < 0.01; * = *p* < 0.05; and *t* = 0.05 ≤ *p* ≤ 0.10. 

## 3. Results

### 3.1. Animal Health

Three animals from the BF treatment were removed before the end of the study, two of them due to lameness problems and one due to an accident. One animal from the C treatment was also removed due to lameness problems.

### 3.2. Intake, Performance, and Carcass Quality

**Figure 1:**
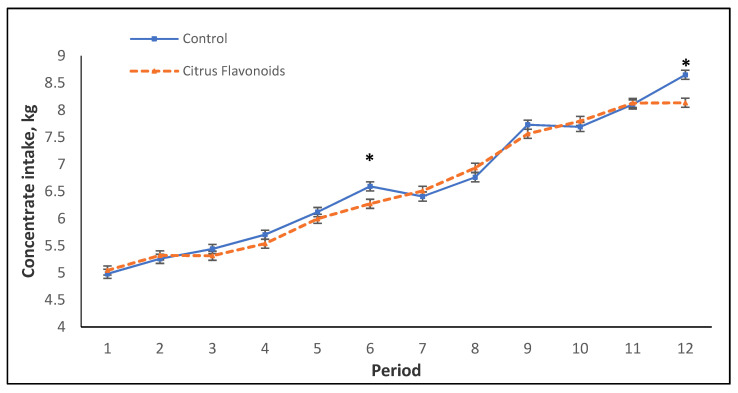
Mean of the concentrate intake during the growing and finishing phases of Holstein bulls fed high-concentrate diets with or without citrus flavonoids supplementation (* = *p* < 0.05).

Although no statistical differences were found in concentrate intake between treatments throughout the study, neither during the growing nor for the finishing phase (Table 2), a significant interaction between treatment and time was found during the whole study (Table 2, Figure 1), as well as during the growing and finishing phases (Table 2). Figure 1 represents the mean of the concentrate intake by period throughout this study. During the growing phase (from period 1 to 8), BF bulls had lower concentrate intake than C bulls, only in period 6 and in the last period of the finishing phase (period 12). An interaction between period and treatment was found for CV of concentrate intake during the finishing phase (Table 2). Thus, the CV was greater for BF than for C bulls during period 9 but, on the contrary, lesser for BF bulls compared with C bulls in period 11, whereas for periods 10 and 12, no differences were found (data not shown). On the other hand, the estimation for straw consumption for the growing phase (0.81 ± 0.065 kg/d and 0.72 ± 0.065 kg/d for C and BF, respectively) and for finishing phase (1.05 ± 0.129 kg/d and 1.08 ± 0.129 kg/d for C and BF, respectively) was not statistically different (*p* = 0.91 and *p* = 0.36, respectively) between treatments either. The performance parameters analyzed throughout the study, such as average daily gain (ADG), final BW, and feed conversion ratio (FCR), did not evince any statistical differences between treatments, neither during the growing phase nor during the finishing phase (Table 2). Regarding carcass quality (data not shown), even though BW before slaughter, HCW, and dressing percentage were not affected by treatment, statistical differences were found for carcass fatness classification. Thus, the C group had a greater (*p* < 0.05) percentage of animals classified with a score of “3” for fatness degree than the BF bulls.

### 3.3. Animal Behavior

Animal behavior data, including general activities along with active behavior, are shown in Table 3 and Table 4 for the growing and finishing phases, respectively.

#### 3.3.1. General Activities 

During the growing and finishing phases, in most of the activities registered, no statistical differences were observed. The proportion of animals ruminating in the BF group was greater (*p* < 0.01) compared with C bulls in the growing and finishing phases, whereas the percentage of animals eating concentrate was greater (*p* < 0.01) for BF compared with C bulls only in the growing phase.

#### 3.3.2. Active Behavior

**Figure 2:**
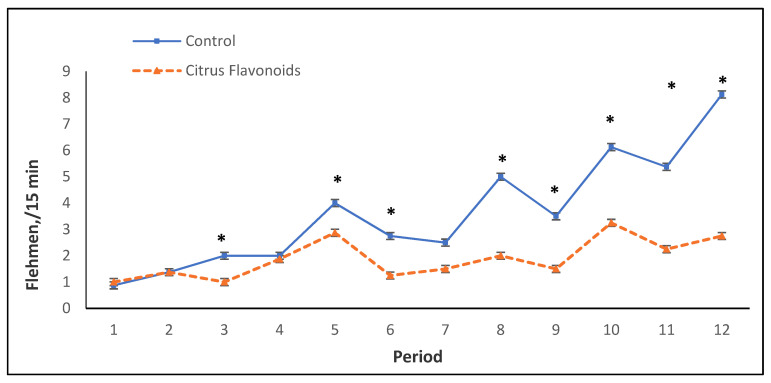
Flehmen every 15 min during the growing and finishing phase of Holstein bulls fed high-concentrate diets with or without citrus flavonoids supplementation (* = *p* < 0.05).

In the growing phase, self-grooming and social behaviors were greater (*p* < 0.001) exhibited by BF compared with C bulls, whereas C bulls exhibited more agonistic behaviors, such as butting (*p* < 0.01), displacement (*p* < 0.001), and chasing (*p* < 0.05), than BF bulls. BF bulls also tended (*p* < 0.10) to perform less fighting, whilst flehmen behaviors were greater (*p* < 0.05) in C bulls compared with BF bulls. Sexual behaviors, such as attempted and completed mounts, were not affected by treatment during this phase. Regarding the finishing phase, self-grooming (*p* < 0.001) and social behaviors (*p* < 0.05) were again greater exhibited by BF than C bulls. During this phase, the C group tended (*p* < 0.10) to perform more oral non-nutritive behaviors compared with BF bulls. Agonistic behaviors, such as fighting (*p* < 0.01), butting (*p* < 0.05), chasing (*p* < 0.05), and displacement (*p* < 0.001), were clearly greater exhibited in this phase by the C group than BF animals. Additionally, C bulls also exhibited a greater amount (*p* < 0.001) of flehmen behaviors (Figure 2, Table 4) and tended (*p* < 0.10) to perform more attempted mounts than BF bulls during this finishing phase (Table 4).

### 3.4. Macroscopic Rumen Evaluation and Liver Abscesses

At the slaughterhouse (Table 5), rumen wall color of the BF bulls was lighter (*p* < 0.05) compared with C bulls (72.86% vs. 50.67% classified as color ≤“3” for BF and C bulls, respectively). No differences between treatments in liver abscesses, baldness regions, and clumped papillae were observed.

### 3.5. Rumen pH and VFA Concentration at Slaughterhouse

The total VFA concentration and pH in the rumen were not affected by treatment (data not shown). The molar proportion of isovalerate was greater (*p* < 0.05) in C bulls compared with BF bulls, whereas the molar proportion of the remaining of VFA analyzed (acetate, propionate, butyrate, valerate, and isobutyrate) were not affected by the treatment (data not shown). Accordingly, the acetate to propionate ratio was also not affected by treatment (data not shown).

### 3.6. Expression of Genes in the Rumen Epithelium

**Figure 3:**
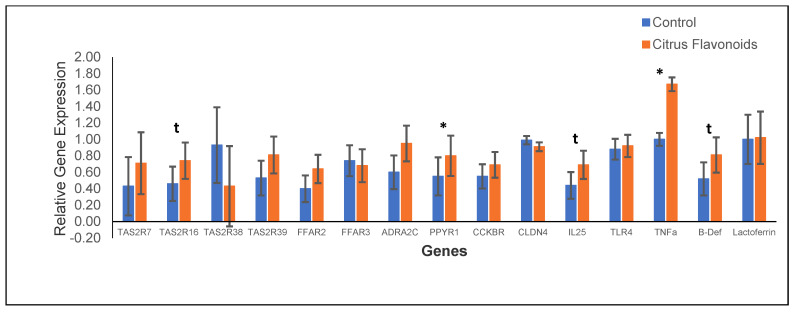
Gene expression in rumen epithelium of Holstein bulls fed high-concentrate diets with or without citrus flavonoids supplementation (* = *p* < 0.05 and t = 0.05 ≤ *p* ≤ 0.10). TAS2R7: *Bitter taste receptor 7*; TAS2R16: *Bitter taste receptor 16*; TAS2R38: *Bitter taste receptor 38*; TAS2R39: *Bitter taste receptor 39*; FFAR2: *Free fatty acid receptor 3 (gpr41)*; FFAR3: *Free fatty acid receptor 2 (gpr43)*; ADRA2C: *Alpha 2-adrenergic receptors subtype C*; PPYR1: *Pancreatic polypeptide receptor 1*; CCKBR: *Cholecystokinin receptor 4*; IL-25: *Interleukin-25*; TLR4: *Pattern recognition receptors, like Toll-like receptor 4*; TNFa: *Tumor necrosis factor alpha*; B-Def: *Beta-defensin*. The values presented herein correspond to back-transformed means; however, SEM corresponds to the ANOVA analyses using log-transformed data.

After RNA extractions, the quality of RNA was acceptable to proceed to qPCR, the ratio of A260/A280 being 1.8–2.0 and that of A260/230 being 2.0–2.2. The results of the relative gene expression in the rumen epithelium are showed in Figure 3. The supplementation with citrus flavonoids only affected the expression of the TAS2R16, which tended (*p* < 0.10) to be greater expressed in BF bulls than in C bulls, whereas the remaining TAS2R analyzed (TAS2R7, TAS2R16, TAS2R38, and TAS2R38) were not affected by treatment. Regarding the relative expression of receptors related to the neurotransmitter signaling, only ppyr1 differed among treatments, being greater (*p* < 0.05) expressed for BF bulls than for C bulls. Additionally, the relative expression of the receptors related to inflammation, such as cytokine IL-25 and β-defensin, also tended (*p* < 0.10) to be greater expressed in BF bulls compared with C bulls, whereas TNFa was greater (*p* < 0.05) expressed in BF bulls than in C bulls. 

## 4. Discussion

In this study, supplementation with citrus flavonoids throughout the fattening period did not affect performance parameters, such as the ADG, final BW, and FCR of bulls. In a previous study carried out with a single-space feeder [4], a reduction in the percentage of large meal sizes along with a reduction in concentrate intake was observed, and this probably negatively affected the final BW of bulls. In the present study, the use of a multispace feeder to feed the animals may have allowed bulls supplemented with flavonoids to visit the feeder more frequently, allowing them to consume similar amounts of concentrate to the control animals in most of the fattening periods (Figure 1), as observed by Paniagua et al. [5]. This would be supported by the visual scan procedure data, as BF bulls occupied more time at the concentrate feeder than C bulls during the growing phase (Table 3). Interestingly, throughout this study, BF bulls did not devote more time to eating straw, and no differences among treatments have been found for straw consumption. Conversely, when a single-space feeder was used [4], a greater occupancy of the straw feeder was observed in bulls supplemented with citrus flavonoids. In agreement with Paniagua et al. [5], the present study results may reinforce the hypothesis that when flavonoids are supplemented and bulls are fed with a single-space feeder, animals may redirect their behavior to eat straw when they cannot access the concentrate feeder. 

As mentioned previously, in concentrate intake, an interaction between treatment and time was observed throughout the study. Figure 1 represents the mean of the concentrate intake by period throughout this study. Only in two periods was a statistical decrease in concentrate intake observed in BF bulls compared with C bulls. From period 7 to the end of the study, an erratic behavior for concentrate intake (greater CV) was observed, especially for C bulls. In this study, bulls were around 7 months old between periods 6 and 7, coinciding with the onset of puberty, during which an increase of production and secretion of testosterone occurs [16,17]. Figure 2 and Figure 3 illustrate flehmen and complete mounts by period, respectively. C bulls clearly and steadily increased the number of flehmen behaviors and tended to exhibit a greater number of attempted mounts from period 7 until the end of the study compared with BF bulls. How this modulation of sexual behaviors by citrus flavonoid supplementation occurs and if this behavior modulation is related to the more erratic concentrate intake observed in C bulls after puberty are not known.

As observed in previous studies [4,5], when supplementing bulls with citrus flavonoids, agonistic, sexual, and oral non-nutritive behaviors were reduced especially during the finishing phase. Oral non-nutritive behaviors are abnormal oral stereotypic behaviors in ungulates. These behaviors may originate due to a digestive dysfunction or a reduction of time devoted to chewing (eating and ruminating) behavior [18]. Reinforcing the idea that non-nutritive oral behaviors could be related to chewing activity, in the present study, during the finishing phase, BF bulls tended to perform less oral non-nutritive behaviors, and simultaneously, these bulls had a greater ruminating activity compared with C bulls. Additionally, rumen wall color was lighter for bulls supplemented with citrus flavonoids, although pH at the slaughterhouse was not affected by treatment. Thus, summarizing the present and previous studies [4,5], it could be hypothesized that supplementation with citrus flavonoids could reduce these oral non-nutritive behaviors by directly improving rumen health (avoiding a digestive dysfunction) or increasing ruminating activity. Currently, as discussed in the previous study [5], mechanisms involved in the gut–brain crosstalk, inflammation (digestive dysfunction) being a key player, could be involved in animal behavior regulation. To further understand if those mechanisms could potentially be involved in behavior modulation, in the present study, the gene expression in the rumen epithelium of some previously described mechanisms was analyzed. For example, inflammation can decrease serum serotonin concentrations, and serotonin is the neurotransmitter known to modulate mood [19] and agonistic behavior [20]. Contrary to expectations, in the current study, citrus flavonoid supplementation increased the relative gene expression of molecules related to inflammation in the rumen epithelium, such as TNFα, cytokine IL-25 (tendency), and β-defensin. Moreover, naringin could act as an antioxidant molecule [21], explaining the lighter color of the rumen wall in BF bulls. Consequently, these results would be in contradiction, necessitating further investigation to discern whether citrus flavonoids supplementation might modulate animal agonistic and sexual behaviors through rumen inflammation mechanisms, or if this modulation takes place beyond the rumen, probably in the intestine, as suggested for monogastric species. 

Otherwise, as previously proposed in our studies [4,5], eating and animal (social and sexual) behaviors would be interrelated, and increasing the time devoted to eating might reduce agonistic and abnormal behaviors in animals [22]. In the current study, when bulls were supplemented with citrus flavonoids, they devoted more time to eating concentrate or performed greater ruminating activity, which could have led to the reduction in agonistic and sexual interactions observed during the visual scan procedure. Thus, we could hypothesize that citrus flavonoids supplementation reduced agonistic and sexual interactions by increasing the time devoted to performing eating behaviors, such as ruminating or eating concentrate.

As discussed in our previous study [5], a possible mode of action of citrus flavonoids to regulate eating behavior could be related to taste receptors (chemosensory transduction). Focusing on the digestive tract, these taste receptors would regulate gut hormones and the release of neurotransmitters, as well as nutrients uptake, being involved in hunger and satiety regulation [23] In fact, bitter chemicals would activate the release of different anorexigenic hormones and peptides in the gastrointestinal tract; some examples of these molecules are ghrelin (orexigenic), cholecystokinin (cck), neuropeptide Y (npy), and peptide YY (pyy) [23,24,25]. 

Citrus flavonoids, and especially naringin, are responsible for the typical bitter taste of citrus fruits, thus being able to activate the TAS2R family [26,27]. In the present study, only TAS2R16 were affected by treatment, and a tendency in increasing the relative gene expression in BF bulls was observed. This TAS2R16 has been described as the bitter receptor for the phytonutrient β-glucopyranosides, which are very ubiquitous in nature [28,29]. In fact, this higher gene expression of TAS2R16 in BF bulls might be related to the naringin content of the citrus flavonoid extract supplemented, as naringin is a glycosylated flavanone [30]. Furthermore, our results have also shown greater gene expression for ppyr1 in bulls supplemented with flavonoids, which acts as a npy and pyy receptor [31]. Thus, npy and pyy are peptides released by TAS2R after a bitter stimulus, and whereas pyy is considered an anorexigenic hormone, npy has been reported as a collateral inhibitor for sweet taste cells when bitter taste cells are stimulated in taste buds [23,25]. Although deeper research is needed, these results could be related to the reduction in meal size observed in bulls when citrus flavonoids were supplemented in the concentrate [4] and the reduced feed intake observed in BF bulls in the present study in some periods. In fact, the possible functions of these TAS2R in rumen epithelium have not been studied, but with our results, it could be hypothesized that citrus flavonoids act in some TAS2R in the rumen epithelium of bulls, modifying the released peptides involved in hunger and satiety and, consequently, modulating the eating patterns of these animals. 

Hassan et al. [32], reviewing the use of mulberry leaf biomass and their flavonoid content on ruminant production, describe that rumen microbial activity increases polymeric flavonoids bioavailibitity by the removal of the sugar group from aglycone. As discussed by Paniagua et al. [5], naringin is a glycosylated flavanone which is deglycosylated by ruminal microflora, as Butyrivibrio spp, into naringenin [33,34]. Thus, naringenin is completely the opposite of naringin from the perspective of taste, acting as a potent bitter masking molecule. That could be a key point of the supplementation of these citrus flavonoids because changes in ruminal fermentation could determine the effects observed when these flavonoids are supplemented. Thus, concentrate presentation (pellet vs. meal) that modulate the eating patterns of the animals and rumen fermentation [6] might affect naringin ruminal metabolism and its effects in bulls as well. In the present study, concentrate has been fed in pellet form, and the results obtained by supplementing citrus flavonoids have been less pronounced compared with a previous study when concentrate was fed in meal form [5]. When compared with the results of our previous study performed with concentrate in meal form [5], in the current study, ruminal VFA and pH were not affected, and the expression of genes in rumen epithelium was less modified than in the previous study. Pelleting the concentrate increases starch gelatinization and reduces particle size, but the hardness of the pellet also affects the accessibility of ruminal bacteria to the nutrients [35]. Therefore, when concentrate supplemented with citrus flavonoids was fed in pellet form, it probably affected ruminal deglycosylation of naringin to naringenin by reducing the accessibility of the ruminal bacteria to the naringin contained within the pellet. Consequently, this process would have taken more time compared with concentrate fed in meal form, probably slowing down naringenin synthesis. On the other hand, if the eating rate is greater for pellets compared with concentrate in meal form [7], a greater quantity of concentrate in a shorter time would arrive to the rumen for fermentation. Thus, these factors might have limited the transformation in the rumen of naringin to naringenin.

Finally, Paniagua et al. [5] found lesser molar percentage of propionate and greater acetate in ruminal liquid samples when bulls fed high-concentrate diets in meal form were supplemented with flavonoids, affecting the acetate to propionate ratio as well. In the current study, the ruminal fermentation parameters analyzed just after slaughtering did not give evidence of any differences among treatments, so propionate, acetate, and total VFA were not affected. Also, pH was similar among treatments. These differences between the present study and Paniagua et al. [5] feeding the same BF doses evince that concentrate presentation, pellet or meal, affect the impact of citrus flavonoids on ruminal fermentation and, consequently, on VFA molar percentages. On the other hand, in the present study, the relative gene expression in ruminal epithelium of ffar2 and ffar3 is consistent with the results obtained for VFA in ruminal liquid, and no differences were observed among treatments for these nutrient-sensing receptors [36].

## 5. Conclusions

The present study reinforces that the supplementation with flavonoids extracted from *Citrus aurantium* reduces agonistic interactions and oral non-nutritive behaviors in bulls fed high-concentrate diets. The concentrate presentation form seems to affect the mechanisms whereby flavonoids act. Further research is needed to fully understand if the modified expression of genes in the rumen epithelium related to inflammation and nutrient sensing is involved in the mode of action of these flavonoids.

## Figures and Tables

**Table 1:** Ingredient and nutrient composition of the dietary concentrates.

Item		
Ingredient, g/kg	Growing	Finishing
Corn grain meal	399.7	450.9
Barley grain meal	179.8	155.5
Dried distillers grains	179.8	150.2
Wheat	109.7	110.3
Beet pulp	73.9	80.2
Palm oil	20.0	25.0
Calcium carbonate	15.5	12.8
Urea	8.0	4.0
Sodium bicarbonate	5.0	4.0
Dicalcium phosphate	3.6	3.1
Vitamin premix	3.0	2.0
Salt	2.0	2.0
Nutrient, per kg DM		
ME, Mcal	3.30	2.97
CP, g	157	123
Ether extract, g	58	54
Ash, g	56	44
NDF, g	178	151
Non-fiber carbohydrates, g	550	628

**Table 2:** Performance and concentrate intake for growing and finishing phases, and for the whole study in Holstein bulls fed high-concentrate diets supplemented with citrus flavonoids.

	Treatment ^1^				*p*-Value *^2^*	
Item	Control	BF	SEM	T	Time	T × Time
Growing phase						
Initial age, d	137.3	137.3	1.81	ns		
Final age, d	259.3	249.3	1.74	ns		
Initial BW, kg	182.8	183.2	7.53	ns		
Final BW (112 d of study)						
Mean, kg	344.5	346.3	7.72	ns		
CV, %	8.1	7.9	0.45	ns		
ADG, kg/d						
Mean, kg/d	1.45	1.45	0.018	ns	***	ns
CV, %	24.6	26.8	2.18	ns	t	ns
Concentrate DM intake						
Mean, kg/d	5.9	5.8	0.09	ns	***	*
CV, %	13.8	13.2	0.76	ns	***	ns
FCR, kg/kg	4.1	4.1	0.05	ns	**	ns
Finishing Phase						
Initial age, d	259.3	249.3	1.74	ns		
Final age, d	306.3	306.3	1.74	ns		
Initial BW, kg	344.6	346.3	7.72	ns		
Final BW (112 d of study),						
Mean, kg	433.9	435.9	7.60	ns		
CV, %	7.9	7.1	0.59	ns		
ADG, kg/d						
Mean, kg/d	1.56	1.55	0.026	ns	ns	ns
CV, %	33.6	32.6	3.23	ns	ns	ns
Concentrate DM intake						
Mean, kg/d	8.0	7.9	0.11	ns	***	**
CV, %	13.1	12.4	0.99	ns	t	*
FCR, kg/kg	5.2	5.1	0.12	ns	ns	ns
Whole study						
Initial age, d	137.2	137.3	1.81	ns		
Final age, d	306.3	306.3	1.73	ns		
Initial BW, kg	182.8	183.2	7.53	ns		
Final BW (168 d of study),						
Mean, kg	433.9	435.9	7.60	ns		
CV, %	7.9	7.1	0.59	ns		
ADG, kg/d				ns		
Mean, kg/d	1.48	1.48	0.018	ns	***	ns
CV, %	27.6	28.8	1.97	ns	**	ns
Concentrate DM intake				ns		
Mean, kg/d	6.6	6.5	0.09	ns	***	**
CV, %	13.6	13.0	0.59	ns	***	ns
FCR, kg/kg	4.5	4.5	0.07	ns	***	ns

^1^ C = nonsupplemented, BF = concentrate supplemented with citrus flavonoids at 0.04%. ^2^ T = treatment effect, Time = time effect (period of 14 d), T × Time = treatment by time interaction effect. *** = *p* < 0.001; ** = *p* < 0.01; * = *p* < 0.05; and t = 0.05 ≤ *p* ≤ 0.10.

**Table 3:** General activities (%) and social behavior (times/15 min) for growing phase in Holstein bulls fed high-concentrate diets supplemented with citrus flavonoids.

Item	Treatment ^1^			*p*-Values ^2^		
	Control	BF	SEM ^3^	T	Time	T × Time
General Activity, %						
Standing	78.46	77.79	0.058	ns	***	ns
Lying	19.66	20.33	0.191	ns	***	ns
Eating concentrate	7.64	9.99	0.060	**	*	ns
Eating straw	11.51	12.81	0.025	ns	***	ns
Drinking	1.55	2.63	0.003	ns	ns	ns
Ruminating	7.66	11.37	0.082	**	*	ns
Social behavior, /15 min						
Self-grooming	19.32	28.95	0.102	***	ns	ns
Social	1.96	4.02	0.126	**	ns	ns
Oral non-nutritive	0.77	0.59	0.049	ns	**	ns
Fighting	9.16	4.91	0.346	0.06	***	ns
Butting	2.30	1.18	0.098	**	***	ns
Displacement	0.464	0.214	0.033	***	ns	ns
Chasing	0.70	0.20	0.071	*	*	0.06
Chasing up	0.16	0.05	0.029	ns	ns	ns
Flehmen	2.21	1.55	0.091	*	**	ns
Attempt to mount	2.21	1.44	0.153	ns	**	ns
Complete mounts	2.55	2.00	0.091	ns	*	ns

^1^ C = non-supplemented, BF = concentrate supplemented with citrus flavonoids at 0.04%. ^2^ T = treatment effect, Time = time effect (measurements every 14 d), T × Time = treatment by time interaction. *** = *p* < 0.001; ** = *p* < 0.01; * = *p* < 0.05; and t = 0.05 ≤ *p* ≤ 0.10. ^3^ SEM = standard error of the means of the log-transformed data (general activity) or root-transformed data (social behavior).

**Table 4:** General activities (%) and social behavior (times/15 min) for finishing phase in Holstein bulls fed high-concentrate diets supplemented with citrus flavonoids.

Item	Treatment ^1^			*p*-Values ^2^		
	Control	BF	SEM ^3^	T	Time	T × Time
General Activity, %						
Standing	67.14	66.41	0.080	ns	**	ns
Lying	32.86	32.93	0.168	ns	**	ns
Eating concentrate	6.27	8.10	0.060	ns	ns	ns
Eating straw	7.86	8.67	0.065	ns	***	ns
Drinking	2.14	2.54	0.004	ns	ns	ns
Ruminating	8.12	14.03	0.072	**	ns	ns
Social behavior, /15 min						
Self-grooming	10.20	16.90	0.150	***	***	ns
Social	3.85	5.93	0.210	*	ns	ns
Oral non-nutritive	1.38	0.78	0.067	t	**	ns
Fighting	8.85	3.85	0.256	**	**	ns
Butting	4.18	1.60	0.214	*	ns	ns
Displacement	1.00	0.15	0.070	***	ns	ns
Chasing	0.56	0.08	0.050	*	ns	ns
Chasing up	0.23	0.05	0.032	ns	ns	ns
Flehmen	5.63	2.35	0.079	***	**	ns
Attempt to mount	1.45	0.10	0.228	t	t	ns
Complete mounts	2.80	1.58	0.165	ns	0.06	ns

^1^ C = non-supplemented, BF = concentrate supplemented with citrus flavonoids at 0.04%. ^2^ T = treatment effect, Time = time effect (measurements every 14 d), T x Time = treatment by time interaction. *** = *p* < 0.001; ** = *p* < 0.01; * = *p* < 0.05; and t = 0.05 ≤ *p* ≤ 0.10. ^3^ SEM = standard error of the means of the log-transformed data (general activity) or root-transformed data (social behavior).

**Table 5:** Macroscopical observations of the rumen and liver at slaughterhouse of Holstein bulls fed high-concentrate diets supplemented with citrus flavonoids.

	Treatment ^1^		*p*-Value ^2^
Item	C	BF	
Color of the rumen ^3^			*
2	2.67	2.86	
3	48.00	70.00	
4	48.00	24.29	
5	1.33	2.86	
Papillae clumping ^4^			ns
Yes	40.00	44.29	
No	60.00	55.71	
Baldness region ^4^			ns
Yes	40.00	49.33	
No	60.00	50.67	
Liver abscess ^5^			ns
None	90.67	94.29	
A	2.67	1.43	
A-	4.00	2.86	
A+	1.33	-	
Inflammation	1.33	1.43	

^1^ C = non-supplemented, BF = concentrate supplemented with citrus flavonoids at 0.04%. ^2^ T = treatment effect. * = *p* < 0.05. ^3^ Adapted from González et al. [9]: rumen color, 1= white; 5 = black. ^4^ Adapted from Nocek et al. [11]. ^5^ Adapted from Brown et al. [12].
